# The impact of long-term aspirin use on the patients undergoing shoulder arthroplasty

**DOI:** 10.1186/s13018-023-04374-4

**Published:** 2023-11-23

**Authors:** Xiuhua Mao, Chen Liang, Xiaoqin Li, Danping Shi, Qinfeng Yang, Hao Xie, Fangguo Liang, Yuhui Cui

**Affiliations:** 1 School of Health, Dongguan Polytechnic, Dongguan, 523000 Guangdong China; 2 Division of Orthopaedic Surgery, Department of Orthopaedics, Nanfang Hospital, Southern Medical University, Guangzhou, 510515 Guangdong China; 3 Department of Neurosurgery, Nanfang Hospital, Southern Medical University, Guangzhou, 510515 Guangdong China; 4 Department of Plastic and Cosmetic Surgery, Nanfang Hospital, Southern Medical University, Guangzhou, 510515 Guangdong China; 5 Department of Medical Imaging Center, Nanfang Hospital, Southern Medical University, Guangzhou, 510515 Guangdong China

**Keywords:** Shoulder arthroplasty, National Inpatient Sample database, Complication, Aspirin

## Abstract

**Background:**

Although aspirin is increasingly utilized to reduce the event of severe perioperative complications, the effect of long-term aspirin use (L-AU) on perioperative complications in patients undergoing shoulder arthroplasty (SA) has not been well studied. The goal of the present study is to identify the influence of L-AU on perioperative complications in individuals undergoing SA.

**Methods:**

We selected data from the National Inpatient Sample database between 2010 and 2019, to identify adult patients with SA. Patients were subsequently categorized into L-AU and whole non-L-AU cohorts according to the presence of aspirin use. The demographic and comorbidity characteristics were matched using propensity score matching (PSM). The Pearson chi-square test, Wilcoxon rank test and logistic regression were utilized to assess the association of L-AU with perioperative complications.

**Results:**

From 2010 to 2019, a total of 162,418 SA patients satisfied the inclusion criteria, with 22,659 (13.95%) using aspirin on a long-term basis. The vast majority of the patients with pre-existing L-AU were aged 65–74 years, female, White and had Medicare insurance. L-AU before surgery was linked to increased risks of perioperative complications, such as blood transfusion (adjusted odds ratio [aOR]: 1.339), genitourinary disease (aOR: 1.349), acute renal failure (aOR: 1.292), acute myocardial infarction (aOR: 1.494), higher total charge (L-AU vs. the whole non-L-AU vs. matched non-L-AU: $66,727.15 vs. $59,697.08 vs. $59,926.32), and prolonged hospitalization stay (LOS) (aOR: 0.837). However, L-AU was considered a protective factor of acute cerebrovascular disease (aOR: 0.722) and stroke (aOR: 0.725).

**Conclusions:**

Our study is based on the largest open-access all-payer inpatient database, revealing a noteworthy finding of aspirin's protective and adverse impact on different postoperative complications in the US population, such as acute cardiovascular disease, and stroke, etc. Further studies assessing the optimum preoperative aspirin duration and dosage to meet the best benefit quantity for patients with planned joint arthroplasties are suggested.

**Supplementary Information:**

The online version contains supplementary material available at 10.1186/s13018-023-04374-4.

## Background

Shoulder arthroplasty (SA) is a recognized cost-effective method for treating various degenerative and traumatic shoulder joint diseases, with a dramatically increasing incidence from 16.70% in 2011 to 32.60% in 2017 among 100,000 people in the USA per year [1]. With the significant increase in the popularity of shoulder replacement surgery in the USA over the past decade [2], aspirin, as an inexpensive, generic, and widely available antiplatelet drug, has been widely used in SA patients in order to reduce the event of severe perioperative complications [3, 4], such as fever remission, deep-vein thrombosis, stroke, fatal pulmonary embolism, inflammation control, postoperative pain, blood transfusion, cardiovascular disease, gastrointestinal complication, and genitourinary disease, etc. Particularly, accumulative evidence has demonstrated that existing long-term aspirin use (L-AU) could inhibit the aggregation of platelets, and by preventing thrombus formation, the perioperative administration of L-AU may significantly prevent serious vascular complications [5, 6]. However, L-AU poses an increased risk of bleeding among patients undergoing surgeries because of its inhibition to platelet function, although this risk appears to be small [7]. But Mangano et al. report that after coronary bypass surgery, taking aspirin early is safe and is linked to lower mortality rates and ischemic complications including the gastrointestinal tract, brain, heart, and kidneys [8]. Obviously, it is challenging and essential to balance L-AU’s positive and negative impacts on the risk of joint perioperative complications.

Taking the enormous benefits provided with aspirin into account, the popularity and prevalence of regular use of aspirin in the USA has been constantly and tremendously increasing [9]. In fact, patients were advised to stop taking aspirin several days (ranging from 2 to 10 days) before elective cardiac surgery by Society of Thoracic Surgeons, the American Heart Association and American College of Cardiology (AHA/ACC), and the European Association for Cardio-Thoracic Surgery (EACTS), primarily because of worries about perioperative bleeding [10]. Furthermore, forty-five years later, aspirin was recommended to prevent venous thromboembolism after orthopedic surgery by the evidence-based clinical practice guidelines of the American College of Chest Physicians [4]. Additionally, a recent randomized controlled trial by the Major Extremity Trauma Research Consortium (METRC) shows that in patients with limb fractures who have undergone surgical treatment or pelvic or acetabular fractures, using aspirin to prevent thrombosis can reduce the incidence of pulmonary embolism and deep vein thrombosis, as well as reduce the 90-day mortality rate [11]. Therefore, it could be confirmed to a certain degree that a pre-existing aspirin use should be clinically meaningful, partly exerting a prophylactic impact on protecting patients from perioperative adverse events. However, to our knowledge, at present, there is no comprehensive study based on large-scale national database analysis, especially on the incidence rate and perioperative complications of L-AU patients after SA.

Considering the above situation, the purpose of this study is to investigate impact of long-term aspirin use on patient undergoing SA based on a national inpatient sample (NIS). Particularly, this study was attempting to determine the prevalence of L-AU after SA. Moreover, another hypothesis was proposed to identify patient groups who can benefit from preoperative optimization by perioperative risk factors. The incidence, Charlson Comorbidity Index (CCI), type of payer, total charges, length of stay (LOS), patients’ demographics, comorbidities, risk factors, and perioperative complications of L-AU following SA were investigated.

## Materials and methods

### Data source

Data were utilized from the 2010–2019 Nationwide Inpatient Sample (NIS), part of the Healthcare Cost and Utilization Project (HCUP), which was funded by the Agency for Healthcare Research and Quality [12]. As the largest public fully paid inpatient observation database in the USA, this database consists of 20.00% of all discharged patients in the USA, and has approximately 8 million acute inpatients from 1050 hospitals in 44 states every year [13], representing approximately 90.00% of non-profit academic medical centers nationwide [14].

### Study cohort

**Fig. 1:**
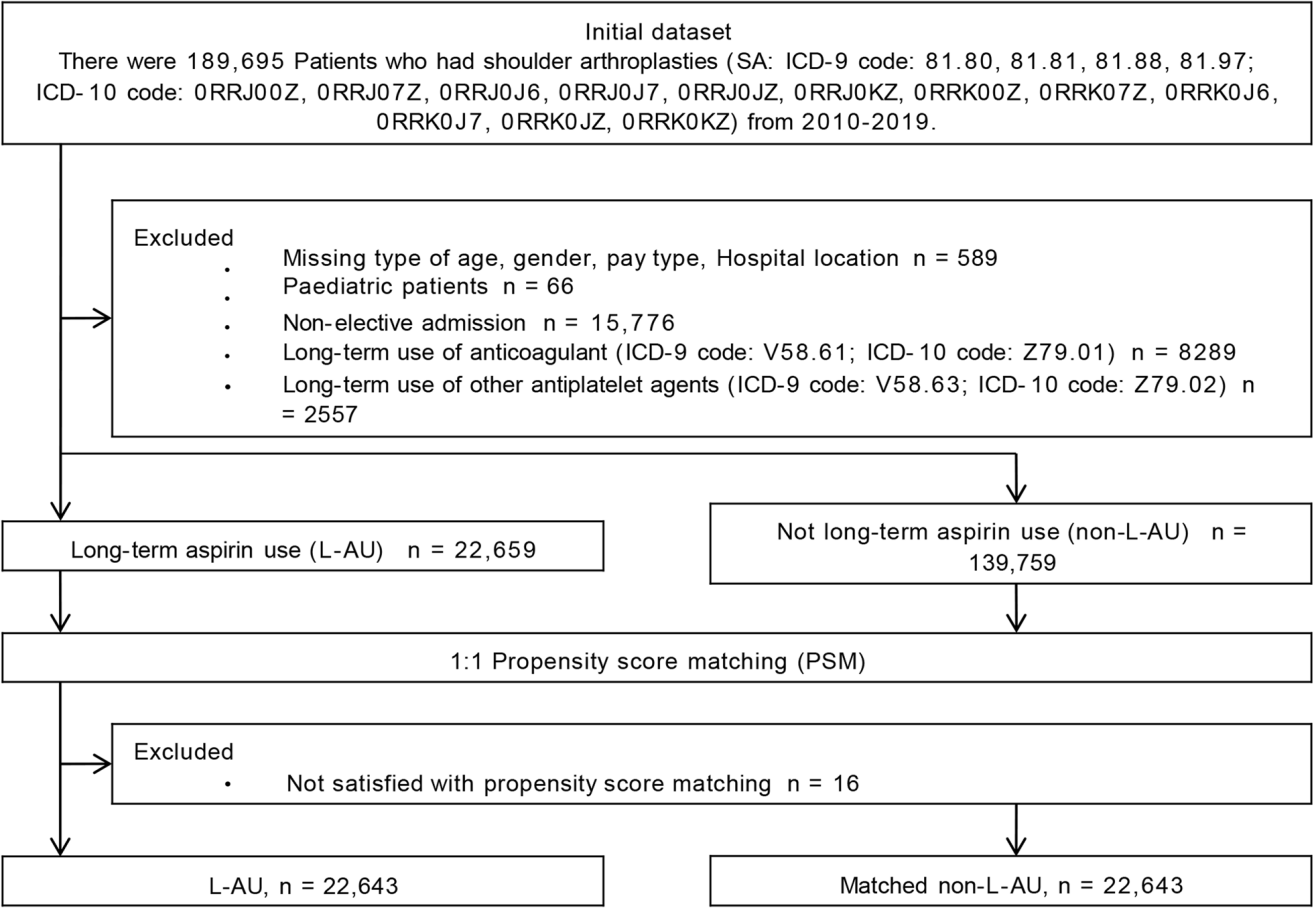
Analysis plan

There were 72,950,400 subjects in the NIS from 2010 to 2019. 189,695 Patients undergoing shoulder arthroplasty were identified based on the procedure codes from International Classification of Disease Clinical Modification version 9 (ICD-9-CM) and the procedure codes from International Classification of Disease Clinical Modification version 10 (ICD-10-CM), (ICD-9 code: 81.80, 81.81, 81.88, 81.97; ICD-10 code: 0RRJ00Z, 0RRJ07Z, 0RRJ0J6, 0RRJ0J7, 0RRJ0JZ, 0RRJ0KZ, 0RRK00Z, 0RRK07Z, 0RRK0J6, 0RRK0J7, 0RRK0JZ, 0RRK0KZ). Out of the extracted dataset of SA patients from 2010 to 2019, 15,842 patients who were under the age of 18 or were hospitalized without selectivity were discarded from the study cohorts. Besides, in order to prevent the interference caused by other antithrombotic drugs, 10,846 patients were excluded with the prolonged use of anticoagulants (ICD-9 code: V58.61; ICD-10 code: Z79.01) or prolonged usage of other antiplatelet drugs (ICD-9 code: V58.63; ICD-10 code: Z79.02). To reduce confusion bias, a 1:1 propensity score matching (PSM) was performed on baseline features, 16 L-AU patients were removed because eligible requirements were not attained (caliper value: 0.01; abandoned rate: 0.05%). Eventually, the final 162,402 patients were divided into two groups: the L-AU cohort (22,643, ICD-9 code: V58.66; ICD-10 code: Z79.82) and non-L-AU cohort (139,759) (Fig. 1).

We evaluated patient and hospital-level characteristics that may affect postoperative morbidity for each cohort. Patients’ characteristics inherently included gender, age subgroups, age, race, pay type, number of comorbidities according to the Charlson Comorbidity Index (CCI), and 29 pre-existing Exlixhauser comorbidities. Hospital characteristics included hospital bed size, hospital teaching status, and hospital location. All the above variables were enrolled in the 1:1 PSM. 16 comorbidity conditions were selected and given a numerical score based on the age-adjusted CCI, and higher score means more comorbidities [15]. Comorbidity conditions and their point values included age (age 41–50 yrs. = 1, age 51–60 yrs. = 2, age 61–70 yrs. = 3, age 71 yrs. = 4), peptic ulcer disease (1), congestive heart failure (1), peripheral vascular disorders (1), other neurological disorders (1), diabetes without complications (1), rheumatoid arthritis/collagen vascular diseases (1), chronic pulmonary disease (1), renal disease (2), diabetes with complications (2), liver disease (2), psychosis (2), solid tumor without metastasis (2), lymphoma (2),  paralysis  (2), AIDS/HIV (2), and metastatic cancer (2) (Table 1).

**Table 1:** Demographic and comorbidities characteristic of study cohorts

Demographic/comorbidity	Study cohorts					
	L-AU^a^ N = 22,643 %	Whole non-L-AU N = 139,759 %	*p* value	Matched non-L-AU N = 22,643 %	*p* value	SMD^b^ after Matching (L-AU & matched non-L-AU)
Gender (%)			< 0.001		0.652	
Male	48.60	42.40		48.30		0.005
Female	51.40	57.60		51.70		0.005
Age (Mean ± SD)^c^	71.11 ± 8.41	68.49 ± 10.01	< 0.001	71.15 ± 8.48	0.994	0.009
Age subgroup (%)			< 0.001		0.433	
18–44	0.30	1.80		0.30		
45–54	2.70	6.50		2.50		0.017
55–64	17.20	22.90		17.60		0.001
65–74	44.30	40.40		44.30		0.002
≥ 75	35.40	28.40		35.40		0.007
Race (%)			< 0.001		0.969	
White	85.00	82.80		85.20		
Black	3.80	4.40		3.90		0.001
Hispanic	2.70	3.80		2.60		0.008
Asian or pacific islander	0.40	0.50		0.40		0.002
Native American	0.30	0.40		0.30		0.001
Other	1.40	1.60		1.40		0.010
Missing	6.30	6.60		6.30		0.004
Pay type (%)			< 0.001		0.806	
Medicare	75.00	67.20		75.20		
Medicaid	2.00	3.30		2.00		0.006
Private insurance	19.1	24.50		19.10		0.009
Self-pay	0.30	0.50		0.30		0.008
No charge	0.00	0.10		0.00		0.006
Other	3.60	4.50		3.40		0.010
Hospital bed size (%)			0.001		0.680	
Small	27.20	26.40		26.90		
Medium	26.60	26.10		26.90		0.010
Large	46.20	47.50		46.20		< 0.001
Hospital location (%)			0.202		0.716	0.012
Rural	91.10	91.30		91.20		
Urban	8.90	8.70		8.80		
Hospital teaching status (%)			< 0.001		0.791	0.005
Teaching	64.10	59.20		64.00		
Non-teaching	35.90	40.80		36.00		
Pre-existing comorbidity (%)						
Hypertension	78.40	64.60	< 0.001	78.50	0.784	0.003
Obesity	22.40	17.50	< 0.001	22.80	0.357	< 0.001
Uncomplicated diabetes mellitus	19.90	15.90	< 0.001	20.10	0.557	0.012
Hypothyroidism	18.50	16.60	< 0.001	18.30	0.680	0.004
Chronic lung disease	19.40	18.40	< 0.001	19.70	0.400	0.002
Depression	19.60	15.70	< 0.001	16.70	0.538	0.009
Deficiency anemia	4.00	4.50	0.003	3.90	0.549	0.004
Fluid and electrolyte disorders	5.60	5.30	0.074	5.70	0.555	0.004
Renal failure	8.20	5.80	< 0.001	8.20	0.732	0.007
Valvular disease	5.00	3.10	< 0.001	5.20	0.380	0.005
Other neurological disorders	2.70	3.10	0.001	2.60	0.681	0.001
Rheumatoid arthritis	4.90	5.30	0.014	5.20	0.133	0.006
Peripheral vascular disorders	4.10	2.30	< 0.001	3.90	0.144	0.011
Congestive heart failure	4.60	3.10	< 0.001	4.50	0.804	0.005
Complicated diabetes mellitus	7.50	3.70	< 0.001	7.20	0.172	0.012
Coagulopathy	1.20	1.30	0.231	1.30	0.866	0.003
Psychoses	2.20	2.40	0.050	2.10	0.383	0.004
Chronic blood loss anemia	0.40	0.50	0.463	0.40	0.516	0.011
Pulmonary circulatory disease	1.10	0.80	< 0.001	1.10	0.529	< 0.001
Alcohol abuse	1.20	1.30	0.245	1.20	0.634	0.014
Liver disease	1.60	1.80	0.056	1.70	0.421	0.007
Solid tumor without metastasis	0.60	0.60	0.667	0.70	0.228	0.001
Drug abuse	0.70	0.80	0.015	0.60	0.816	0.001
Lymphoma	0.20	0.30	0.340	0.30	0.779	0.004
Paralysis	0.30	0.40	0.011	0.30	0.266	0.005
Weight loss	0.20	0.30	0.089	0.20	0.844	0.004
Metastatic cancer	0.20	0.20	0.164	0.20	1.000	0.007
Peptic ulcer disease excluding bleeding	0.20	0.20	0.013	0.30	0.703	0.005
Acquired immune deficiency syndrome	0.10	0.20	0.014	0.10	0.622	0.003
Charlson comorbidity index subgroup (%)			< 0.001		0.648	
0	0.10	0.60		0.10		
1	0.50	2.20		0.40		0.019
2	4.50	9.40		4.40		0.009
≥ 3	94.90	87.70		95.10		0.014

^a^L-AU: long-term aspirin use

^b^SMD: standardized mean difference

^c^SD: standard difference

### Outcomes

Perioperative complications after shoulder arthroplasty were searched from the database and the detailed items are listed (Table 2). ICD-9 diagnosis codes and ICD-10 diagnosis codes (Additional file 1) were used to identify any complication, blood transfusion, periprosthetic joint infection, dislocation of prosthetic joint, hemorrhage/seroma/hematoma, urinary tract infection, acute renal failure, thrombocytopenia, acute postoperative pain, respiratory disease, genitourinary disease, pneumonia, gastrointestinal complication, convulsion, deep venous thrombosis, wound infection, pulmonary embolism, acute myocardial infarction, peripheral vascular disease, postoperative delirium, septicemia, acute cerebrovascular disease, cardiac arrest, postoperative shock, gastrointestinal bleeding, stroke, and death. In addition, “any complication” was defined as the patient having at least one complication. When patients were in pain within the initial 72 h after surgery, acute postoperative pain was recorded. As NIS collected inpatient databases, the term "perioperative" specifically applied to the period from patients’ hospitalization to discharge in our study. Finally, the total cost and length of stay (LOS) during the hospitalization were used to quantify resource consumption. Only when the total cost and the length of stay (LOS) outrun 75.00%, respectively, were they considered to be prolonged stays and higher costs.

**Table 2:** Perioperative complications

Perioperative complication	Study cohorts				
	L-AU^a^	Whole non-L-AU	*p* value	Matched non-L-AU	*p* value
	N (%)	N (%)		N (%)	
Any complication^b^	3603 (15.91)	19,639 (14.05)	< 0.001	3631 (16.04)	0.719
Blood transfusion	505 (2.23)	4062 (2.91)	< 0.001	671 (2.96)	< 0.001
Periprosthetic joint infection (PJI)	112 (0.49)	619 (0.44)	0.281	120 (0.53)	0.598
Dislocation of prosthetic joint	260 (1.14)	1461 (1.05)	0.161	257 (1.18)	0.894
Hemorrhage/seroma/hematoma	36 (0.15)	268 (0.19)	0.290	42 (0.19)	0.497
Urinary tract infection (UTI)	286 (1.26)	2032 (1.45)	0.025	328 (1.45)	0.088
Acute renal failure (ARF)	323 (1.43)	1993 (1.43)	0.996	415 (1.83)	0.001
Thrombocytopenia	233 (1.03)	1373 (0.98)	0.511	211 (0.93)	0.294
Acute postoperative pain	657 (2.90)	3665 (2.62)	0.015	590 (2.61)	0.054
Respiratory disease	88 (0.39)	521 (0.37)	0.717	93 (0.41)	0.710
Genitourinary disease	577 (2.55)	4004 (2.86)	0.008	771 (3.41)	< 0.001
Pneumonia	77 (0.34)	500 (0.36)	0.678	92 (0.41)	0.248
Gastrointestinal complication	22 (0.09)	86 (0.06)	0.054	16 (0.07)	0.330
Convulsion	89 (0.39)	529 (0.38)	0.742	74 (0.33)	0.239
Deep venous thrombosis (DVT)	15 (0.07)	119 (0.09)	0.358	14 (0.06)	0.853
Wound infection	9 (0.04)	63 (0.05)	0.724	7 (0.03)	0.617
Pulmonary embolism (PE)	21 (0.09)	155 (0.11)	0.441	28 (0.12)	0.317
Acute myocardial infarction (AMI)	59 (0.26)	379 (0.27)	0.775	88 (0.39)	0.017
Peripheral vascular disease	971 (4.29)	3545 (2.54)	< 0.001	916 (4.05)	0.196
Postoperative delirium (POD)	110 (0.48)	616 (0.44)	0.346	118 (0.52)	0.595
Septicemia	15 (0.07)	138 (0.10)	0.139	14 (0.06)	0.853
Acute cerebrovascular disease (ACD)	211 (0.93)	697 (0.49)	< 0.001	153 (0.68)	0.002
Cardiac arrest	11 (0.05)	72 (0.05)	0.856	18 (0.08)	0.194
Postoperative shock	4 (0.02)	48 (0.03)	0.193	10 (0.04)	0.109
Gastrointestinal bleeding (GI bleeding)	12 (0.05)	59 (0.04)	0.472	14 (0.06)	0.695
Stroke	209 (0.92)	691 (0.49)	< 0.001	152 (0.67)	0.003
Death	9 (0.04)	69 (0.04)	0.540	13 (0.06)	0.394

^a^L-AU: long-term aspirin use

^b^Any complication: defined as at least one complication observed in Table 3 that occurred in a patient

### Statistical analysis

**Fig. 2:**
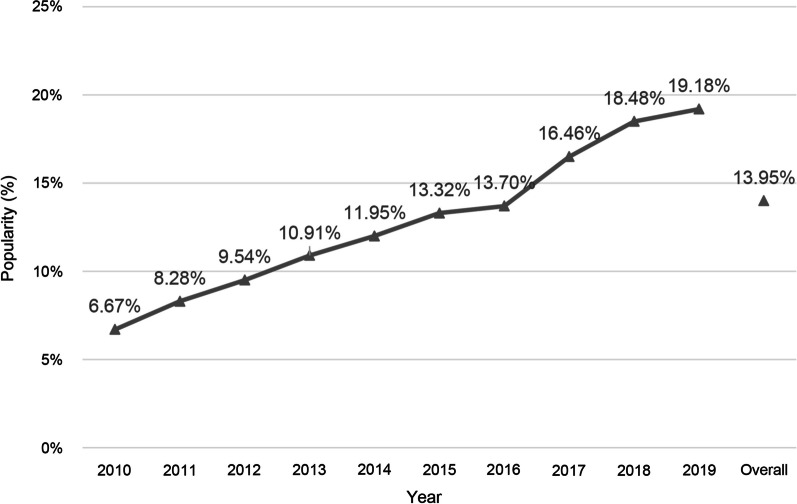
The annual popularity of pre-existing long-term aspirin use in patients undergoing shoulder arthroplasty between 2010 and 2019 in the United States

The overall occurrence of L-AU among patients undergoing SA in the USA from 2010 to 2019 was calculated in the National Inpatient Sample database (Fig. 2).

In order to test the independent influence of L-AU on perioperative complications and avoid the deviation between non-L-AU and L-AU cohorts, we conducted a 1:1 PSM cohort analysis to match the demographics and comorbidity characteristics of patients between non-L-AU and L-AU cohorts (Table 1). The matching caliper value in PSM is 0.01, and the confounding factors for matching include age, age subgroup, race, gender, hospital teaching status, bed size, hospital location, Charlson comorbidities index subgroup, and previously existing Exlihauser comorbidities. After PSM, 22,643 matched non-L-AU and 22,643 L-AU patients (16 patients were discarded, with a rejection rate of 0.05%) were included in the following analysis. Due to all p-values above 0.050, there was no statistically significant difference in demographic and comorbidity characteristics between the L-AU and matched perioperative complications group. In addition, the standardized mean difference between L-AU and matched non-L-AU queues is less than 0.10, indicating that after PSM, no matching covariates changed their distribution (Table 1).

In order to demonstrate the statistical variance between L-AU and matched non-L-AU cohorts, Wilcoxon rank tests were performed on continuous variables and Pearson chi square tests were calculated on categorical variables. All tests were considered statistically significant, with *p* < 0.050. To evaluate the relationship between L-AU and perioperative complications, univariate and multivariate logistic regression models were used (Tables 2 and 3). The 95% confidence intervals (CI) and odds ratio (OR) were estimated for the whole non-L-AU group and the non-L-AU group which served as the control groups. The unadjusted odds ratios (uOR) and the multivariable—adjusted odds ratios (aOR) was provided, respectively. An alpha level of *p* < 0.050 was used to determine statistical significance, which has been utilized by other NIS researches [16].

**Table 3:** Unadjusted/multivariable-adjusted odds ratios of perioperative complications^a^

Perioperative complication	Whole non-L-AU as controls			Matched non-L-AU as controls		
	Unadjusted OR (uOR)	(Wad 95%CI)	*p* value	Adjusted OR (aOR)	(Wad 95%CI)	*p* value
Any complication^b^	0.871	0.825–0.920	< 0.001	0.991	0.942–1.042	0.719
Blood transfusion	1.465	1.322–1.632	< 0.001	1.339	1.191–1.505	< 0.001
Periprosthetic joint infection (PJI)	1.117	0.913–1.367	0.281	0.933	0.721–1.208	0.598
Dislocation of prosthetic join	1.100	0.963–1.256	0.161	1.012	0.851–1.203	0.894
Hemorrhage/seroma/hematoma	0.829	0.585–1.174	0.290	0.857	0.549–1.338	0.497
Urinary tract infection (UTI)	0.867	0.765–0.982	0.025	0.870	0.742–1.021	0.088
Acute renal failure (ARF)	1.000	0.889–1.126	0.996	1.292	1.115–1.496	0.001
Thrombocytopenia	1.048	0.911–1.205	0.511	1.105	0.917–1.333	0.294
Acute postoperative pain	1.110	1.020–1.207	0.015	1.117	0.998–1.250	0.054
Respiratory disease	1.043	0.832–1.308	0.717	0.946	0.706–1.267	0.710
Genitourinary disease	1.247	1.130–1.375	< 0.001	1.349	1.209–1.506	< 0.001
Pneumonia	0.950	0.747–1.209	0.678	0.836	0.618–1.133	0.248
Gastrointestinal complication	1.580	0.989–2.523	0.054	1.375	0.722–2.619	0.330
Convulsion	1.039	0.829–1.301	0.742	1.204	0.884–1.639	0.239
Deep venous thrombosis (DVT)	0.778	0.455–1.331	0.358	1.071	0.517–2.220	0.853
Wound infection	0.882	0.438–1.773	0.724	1.286	0.479–3.453	0.617
Pulmonary embolism (PE)	0.836	0.530–1.319	0.441	0.750	0.426–1.321	0.317
Acute myocardial infarction (AMI)	0.961	0.730–1.264	0.775	1.494	1.073–2.078	0.017
Peripheral vascular disease	0.659	0.605–0.718	< 0.001	1.063	0.969–1.165	0.196
Postoperative delirium (POD)	1.103	0.900–1.351	0.346	0.932	0.718–1.209	0.595
Septicemia	0.671	0.394–1.143	0.139	1.071	0.517–2.220	0.853
Acute cerebrovascular disease (ACD)	0.630	0.537–0.740	< 0.001	0.722	0.586–0.891	0.002
Cardiac arrest	0.943	0.500–1.779	0.856	0.611	0.288–1.294	0.194
Postoperative shock	0.514	0.185–1.426	0.193	0.400	0.125–1.275	0.109
Gastrointestinal bleeding (GI bleeding)	1.256	0.675–2.336	0.472	0.857	0.396–1.853	0.695
Stroke	1.875	1.605–2.190	< 0.001	0.725	0.587–0.894	0.003
Prolonged length of stay (> 75th percentile)	0.889	0.857–0.923	< 0.001	0.837	0.798–0.877	< 0.001
Higher total cost (> 75th percentile)	1.009	0.977–1.041	0.600	0.968	0.928–1.009	0.128

^a^L-AU: long-term aspirin use

^b^Any complication: defined as at least one complication observed in Table 3 that occurred in a patient

No ethical approval was required because the study used a publicly accessible database. All statistical analyses were conducted using SPSS version 25 (IBM Corp., Armonk, NY).

## Results

### Occurrence of L-AU in patients undergoing SA

162,418 patients undergoing SA were retrieved in the NIS database between 2010 and 2019. In general, there were 22,659 cases of long-term aspirin use with an average prevalence of 13.95% (22,659/162,418) (Fig. 2). Significantly, it was discovered that the annual incidence of L-AU increased yearly from 2010 to 2019 (from 6.67 to 19.18%) (Fig. 2).

### Patient demographics and comorbidity characteristics among three study cohorts

Before conducting PSM at a ratio of 1:1, significant differences in demographics and comorbidity between L-AU and the entire non-L-AU cohort were observed. Long-term aspirin patients were more likely to be female, elderly, White and paid with Medicare (*p* < 0.001) (Table 1). L-AU patients had lower likelihood to be hospitalized in a small-bed and non-teaching hospital (*p* < 0.001) (Table 1). Particularly, there was a significant difference in Charlson comorbidity index between L-AU and entire non-L-AU patients, and patients with Charlson comorbidity index ≥ 3 accounted for the largest proportion of both L-AU and whole non-L-AU ones (the whole non-L-AU vs. matched non-L-AU vs. L-AU: 87.70% vs. 95.10% vs.94.90%), which as mentioned previously, illustrated more comorbidities (*p* < 0.001) (Table 1). The incidence of comorbidities in almost half of L-AU patients is significantly higher than that in whole non-L-AU patients apart from deficiency anemia, other neurological disorders, rheumatoid arthritis, coagulopathy, psychoses, chronic blood loss anemia, liver disease, alcohol abuse, lymphoma, paralysis, drug abuse, fluid and electrolyte disorders, solid tumor without metastasis, weight loss, peptic ulcer disease excluding bleeding, metastatic cancer, and acquired immune deficiency syndrome (*p* < 0.001) (Table 1). Notably, the occurrence of uncomplicated diabetes mellitus, obesity, hypertension, depression, renal failure, and complicated diabetes mellitus comprised a significantly higher proportion in L-AU patients in contrast to whole non-L-AU patients outrunning by 13.80%, 4.90%, 4.00%, 3.90%, 3.80%, and 2.40%, respectively (*p* < 0.001) (Table 1). After the PSM by 1:1 ratio, all the confounding variables in Table 1, including gender, age, age subgroups, race, the type of payer, the bed size of hospital, the location of hospital, the teaching status of hospital. 29 pre-existing Exlihauser comorbidities and Charlson comorbidity index subgroups represented no apparent difference between L-AU and matched non-L-AU cohorts as control (*p* > 0.050) and all between-group standardized mean differences were less than 0.100 which were considered balanced (Table 1).

### Adverse effects of L-AU after SA

Noteworthy is that the majority of complications’ incidences were less than 1.00%, apart from any complication (14.10–15.90%), peripheral vascular disease (2.50–4.30%), acute postoperative pain (2.60–2.90%), genitourinary disease (2.50–2.90%), blood transfusion (2.20–2.90%), acute renal failure (1.40–1.80%), urinary tract infection (1.30–1.50%) and dislocation of prosthetic joint (1.00–1.10%) (Table 2). Compared with the whole non-L-AU ones (*p* < 0.010) (Table 2), patients with L-AU had an increased risk of perioperative complications, such as blood transfusion, genitourinary disease, peripheral vascular disease, acute cerebrovascular disease, stroke, and overall perioperative complications. However, L-AU patients were inclined to suffer perioperative complications such as blood transfusion, acute renal failure, genitourinary disease, acute cerebrovascular disease, and stroke (*p* < 0.010), in contrast to matched non-L-AU patients (Table 2).

In relation to isolated effect of L-AU, logistic regression analysis was used to identify assess the association of L-AU with perioperative complications. Between the L-AU and whole non-L-AU groups, patients with L-AU were more likely to have blood transfusion (uOR = 1.465; 95% CI = 1.322–1.632; *p* < 0.001), acute postoperative pain (uOR = 1.110; 95% CI = 1.020–1.207; *p* = 0.015), and stroke (uOR = 1.875; 95% CI = 1.605–2.190; *p* < 0.001), but less likely to have any complication (uOR = 0.871; 95% CI = 0.825–0.920; *p* < 0.001), urinary tract infection (uOR = 0.867; 95% CI = 0.765–0.982; *p* = 0.025), prolonged length of stay (> 75th percentile) (uOR = 0.889; 95% CI = 0.857–0.923; *p* < 0.001), peripheral vascular disease (uOR = 0.659; 95% CI = 0.605–0.718; *p* < 0.001), acute cerebrovascular disease (uOR = 0.630; 95% CI = 0.537–0.740; *p* < 0.001) (Table 3). Consistent with the multivariate adjusted analysis, a significant correlation was found between L-AU and blood transfusion (aOR = 1.339; 95% CI = 1.191–1.505; *p* < 0.001), and prolonged length of stay (> 75th percentile) (aOR = 0.837; 95% CI = 0.798–0.877; *p* < 0.001). While L-AU was a risk factor for acute renal failure (aOR = 1.292; 95% CI = 1.115–1.496; *p* < 0.001), genitourinary disease (aOR = 1.349; 95% CI = 1.209–1.506; *p* < 0.001), acute myocardial infarction (aOR = 1.494; 95% CI = 1.073–2.078; *p* = 0.017), it was noteworthy that patients with L-AU were less likely to have acute cerebrovascular disease (aOR = 0.722; 95% CI = 0.586–0.891; *p* < 0.002) and stroke (aOR = 0.725; 95% CI = 0.587–0.894; *p* = 0.003) (Table 3). Importantly, L-AU was a significant risk factor for acute renal failure (aOR = 1.292) and acute myocardial infarction (aOR = 1.494), but unadjusted ORs demonstrated no significant statistical difference contrarily (Table 3). Furthermore, adjusting OR indicates that L-AU is not associated with an increased risk of postoperative acute pain (aOR = 1.117, *p* = 0.054) and peripheral vascular disease (aOR = 1.063; *p* = 0.196), but unadjusted ORs indicate that L-AU was an important protective element (acute postoperative pain, uOR = 1.485; peripheral vascular disease, uOR = 0.659) (Table 3).

Not surprisingly, L-AU patients are typically discharged earlier compared with non-L-AU and matched non-L-AU patients (L-AU vs. the whole non-L-AU vs. matched non-L-AU: 2.03 vs. 2.07 vs. 2.10 days; *p* < 0.001) (Table 4). Nonetheless, the presence of L-AU resulted in a clear $7,030 increase in total hospital expenses. ($66,727.15 vs. $59,926.32 vs. $59,697.08; *p* < 0.001) (Table 4). Therefore, L-AU increased medical expenses. Accordingly, it was discovered that patients with L-AU were less likely to use private insurance and more likely to pay through Medicare (*p* < 0.001) (Table 1). Furthermore, the number of disease diagnoses (11.06 vs. 8.51 vs. 8.02) shows significant statistical difference among the three cohorts (*p* < 0.001) (Table 4). In this study, L-AU was a separate protective element that reduced LOS (aOR = 0.837). Nevertheless, L-AU patients often have more disease diagnoses than matched non-L-AU and whole non-L-AU patients (11.06 vs. 8.51 vs. 8.02 diagnoses), which may have contributed to their higher total cost of discharge (aOR = 0.968).

**Table 4:** Resource consumption

Resource consumption	Study cohorts				
	L-AU	Whole non-L-AU	*p* value	Matched non-L-AU	*p* value
Length of stay (Days)			< 0.001		< 0.001
75th percentile	1.00–2.00	1.00–3.00		1.00–3.00	
Mean	2.03	2.07		2.10	
Total charge (USDs)			< 0.001		< 0.001
75th percentile	44,358.50–79,451.00	38,608.0–72,378.00		38,492.75–72,714.00	
Mean	66,727.15	59,697.08		59,926.32	
Number of disease diagnoses			< 0.001		< 0.001
Mean	11.06	8.02		8.51	
Number of medical procedures			0.515		0.744
Mean	1.65	1.66		1.66	
Days from admission to surgery			0.033		0.013
Mean	0.05	0.05		0.07	

## Discussion

This study conducted a large-scale health economic analysis on L-AU patients after SA. To our knowledge, this is the first objective study to investigate the impact of L-AU on patients receiving SA from 2010 to 2019. In our study, the prevalence of pre-existing L-AU significantly increased from 6.70% (2010) to 19.20% (2019), indicating that effective use of aspirin is still crucial for effectively reducing the occurrence of these complications, such as arterial and venous thrombosis, nonfatal myocardial infarction, ischemic stroke, acute pain and even colorectal cancer and death, as recommended by prior studies [4, 17–20] (Fig. 2). Multiple factors may account for the steady rise in incidence rates from 2010 to 2019. On the one hand, the expanding proportion of elderly population (sixty-five years of age or older) patients receiving SA in the USA may have contributed to the increase [21]. In the meantime, the targeted population for primary/secondary cardiovascular disease (CVD) prevention is typically the elderly, and a modest dose of aspirin is routinely recommended for individuals aged ≤ 70 years by 2019 guidelines [22, 23]. Particularly, female, advanced age, hypertension, obesity, hypothyroidism, chronic lung disease, depression, uncomplicated diabetes mellitus, peripheral vascular disorders, congestive heart failure, complicated diabetes mellitus, pulmonary circulatory disease, valvular disease, and renal failure were significant feature of L-AU patients in this study, consistent with the risk factors for CVD [24]. On the other hand, large amounts of aspirin have been continuously put into use, such as colorectal cancer and pre-eclampsia prevention methods proposed by the U.S. Preventive Services Task Force (USPSTF) [25, 26]. Overall, the previous study has revealed that aspirin can effectively prevent venous thromboembolism after both total hip arthroplasty and total knee arthroplasty [9]. Moreover, when SA patients undergo traumatic arthroplasty, aspirin’s long-term effect might continue to benefit them, while other cumulative benefits previously provided by L-AU, such as sustained reduction of the burden of oxidative stress, inflammation, and endothelial dysfunction in many aspects, may continue to benefit them.

In our study, first of all, although most L-AU patients have a higher burden of complications, the current research has proved for the first time that pre-existing L-AU will significantly increase the risk of perioperative blood transfusion, acute renal failure, genitourinary system diseases and most other perioperative complications of SA patients. The cause of blood transfusion and acute myocardial infarction may be the reduction of TXA2-dependent platelet activity, a crucial aspect of primary haemorrhage, which is the principal risk of the low-dosage aspirin therapy [27]. Among patients at high risk of cardiovascular disease, observational study [28] and a meta-analysis of randomized trials [29] have illustrated that long term, low-dose aspirin treatment can double the risk of severe extracranial bleeding (mainly upper gastrointestinal bleeding). These serious bleeding complications are substantially more likely to occur in patients over the age of 70. Researchers also suggested that compared to not taking aspirin, taking aspirin almost doubles the risk of gastrointestinal and other extracranial bleeding events [30]. Therefore, before undergoing shoulder arthroplasty, a 24 h dosing interval of low-dose aspirin administration (81 mg per day) is frequently considered to be sufficient to keep TXA2-dependent platelet activation almost completely and persistently suppressed suggested by the Patient-Centric Trial Assessing Benefits and Long-term Effectiveness (ADAPTABLE) Trial [31, 32]. Notably, pre-existing L-AU also serves as a risk factor of genitourinary disease and acute renal failure, in accordance with the former study implying that the long-term use of high-dose aspirin resulted in renal papillary necrosis (RPN) and renal dysfunction [33]. Although low-dose aspirin may be suitable for women who are considered particularly susceptible to early-onset pre-eclampsia, severe enough to require premature delivery [26], previous research findings do not support routine prophylactic or therapeutic antiplatelet therapy during pregnancy for all women with increased risk of preeclampsia or intrauterine growth retardation [34], which is consistent with present study. However, according to Liang FG’s research [35], aspirin is a protective factor for acute renal failure, blood transfusion and genitourinary disease, which is totally contrary to our study. A prophylactical low dose of aspirin is recommended by 2019 ACC/ACH guidelines [22], so maybe long-term use of aspirin could lead in higher risk of bleeding. It is worth noting that renal failure incidence in L-AU patients was slightly higher than non-L-AU patients in the their study, but acute renal failure is showed to be a protective factor, which seems contradictory in their study. Also, their surgical methods for different parts, latest time range and ICD-10 diagnosis methods may mainly contribute to the different outcomes.

In the present study, however, it is worthy of noting that the incidence of acute cerebrovascular disease and stroke in L-AU patients is marginally lower than that in non-L-AU patients. When cyclooxygenase-1 is blocked for a long time, many defense performances of L-AU are caused by various physiological changes, and its advantages may be beneficial results [4]. In brief, the effect of aspirin depends on the inhibition of cox enzyme, a component of the arachidonic acid metabolism pathway that catalyzes the conversion of arachidonic acid to prostaglandin H2 and subsequently produces thromboxane A2 (TXA2) and prostaglandin I2 (PGI2). While PGI2 promotes vasodilation and the suppression of platelet aggregation, TXA2 causes vasoconstriction. Aspirin is thought to have a protective effect against vascular diseases because it mainly affects the acetylation of serine residues in the cox channel and blocks the entry of substrates into the catalytic sites of enzymes in megakaryocytes. Therefore, aspirin can affect the aggregation characteristics of newly generated platelets [36]. The antiplatelet effect of aspirin lasts for 7 to 10 days in line with the lifespan of new platelets. In addition, as our study has shown, reduced length of stays was also one of the advantages of L-AU, consistent with the previous NIS database analysis study [35].

Second, by blocking prostaglandin synthesis and sensitization of pain receptors, aspirin can be used as an analgesic [20], and it has been demonstrated to be beneficial for severe postoperative pain. Contrary to expectations, our research results indicate that compared to the entire non-L-AU group, L-AU patients have a higher risk of acute postoperative pain, so pre-existing L-AU may reduce patients' tolerance to postoperative pain who underwent total knee arthroplasty. Although there have been few previous studies describing this phenomenon, rodent models of inflammatory pain have shown that low-dose aspirin can alleviate escape or avoidance behavior, but cannot alleviate mechanical hyperalgesia [37]. Therefore, we reckon that low-dose aspirin's long-term anti-inflammatory impact may result in hyperalgesia and the increase of pain sensitivity. Further studies should be performed to explore the potential mechanisms and comprehend this phenomenon.

Third, the balance between the potential risk of bleeding and the expected benefits of aspirin remains a focus of clinical attention [27]. Studies have shown that low-dose aspirin has a lower risk of gastrointestinal (GI) bleeding episodes and non-gastrointestinal bleeding episodes (intracranial hemorrhage and hemorrhagic stroke) [28]. Although our study reveals that, from 2010 to 2019, pre-existing L-AU had no association with GI bleeding, thrombocytopenia, or hemorrhage/seroma/hematoma, L-AU was a significant risk factor for blood transfusion. This may be due to the potential bleeding risk caused by the inhibition of platelet aggregation by aspirin, and routine preoperative discontinuation of aspirin is not safe enough for rapid recovery of coagulation function in patients. Consequently, compared to whole non-L-AU patients, the risk of bleeding was at least greater in L-AU patients, which serves as a reminder for the future research to find a safe and appropriate aspirin dose for long-term use.

Fourth, L-AU was identified as a significant and advantageous factor of a shorter length of stay in the present study, despite of the fact that the results indicated that SA patients with L-AU usually have greater disease burden, so they use more medical resources. Similarly, Wayangankar’s study also illustrated that aspirin can shorten hospital stay in patients undergoing Femoral artery transcatheter aortic valve replacement [38].

In summary, pre-existing L-AU is shown to be a significant risk factor for blood transfusion, acute renal failure, genitourinary disease, and acute myocardial infarction. Contrariwise, it serves as a protective factor for acute cerebrovascular disease and stroke. Although there is a risk of bleeding, the pre-accumulated benefits of aspirin may still reduce the risk of perioperative complications and hospital resource consumption. Consequently, the present study proposes an evidence-deficient but justifiable hypothesis that if patients who plan to have surgery in the future can take the best and appropriate dose of aspirin in sufficient time, long-term use of aspirin can better prevent perioperative complications of shoulder joint replacement surgery.

Our study has several important limitations. First, the most suitable duration and dose of aspirin could not be determined when patients are expected to undergo orthopedic surgery. Hence, we could not further determine whether the effects of aspirin dosage and duration have an independent association with perioperative complications. Further research may supplement subgroup analysis of the dose and duration of aspirin intake, which can help determine the ideal dose and duration of L-AU, thereby approaching the maximum benefit risk ratio of L-AU and minimizing perioperative risks. In addition, our results rely on complete data records and accurate coding. However, as the largest management database carrying patient data, the NIS database has been used in many studies, especially those investigating the perioperative consequences of orthopedic surgery patients. Meanwhile, NIS was developed based on the AHRQ Healthcare Cost and Utilization Project (HCUP), which includes administrative and demographic data from 20.00% of inpatient samples in the USA. Since 1988, through collaboration among multiple state wide data organizations, it has been compiled annually to provide data on the utilization rate of all paid healthcare [39]. Therefore, the NIS database has high credibility in the data source. Finally, given that the NIS database does not distinguish between initial hospitalization and subsequent hospitalization, we cannot determine multiple hospitalizations for the same patient. Additionally, the NIS database does not contain information on the drugs that the patients received both within the hospital and outside of it. Nevertheless, considering the large size of our sample, it is not likely that this scenario will have a substantial impact on our findings. Unlike registries, which are known to have strong a referral bias, the NIS database allows a larger, more diverse sampling of the real-world experience. The NIS database, which includes data from 46 states in the United States, is the largest fully paid hospitalization database, making it the best tool for analyzing the trends and consequences of rare disease hospitalizations such as L-AU in the real world [40].

## Conclusions

We found a significant increase in patients experiencing SA with pre-existing L-AU in the United States. Despite patients receiving L-AU therapy, there were no statistically significant differences in the number of medical procedures and the number of days from admission to surgery between patients with long-term and non-long-term use of aspirin. Patients with L-AU tend to have more disease diagnoses and a large rise in the total charge though a noticeable decrease in the length of hospitalization was observed. Pre-existing L-AU was associated with increased risk of perioperative complications, such as blood transfusion, acute renal failure, genitourinary disease and acute myocardial infarction. Nonetheless, pre-existing L-AU was identified as a protective factor for acute cerebrovascular disease and stroke. As the answer the second hypothetical question, patients with acute cerebrovascular disease and stroke can improve their postoperative prognosis by taking aspirin for a long time before shoulder replacement surgery. According to the information we have, our work is the first to demonstrate a significant impact of pre-existing L-AU on SA patients. Therefore, we put forward a hypothesis: if further research can find out the appropriate duration and dose of preoperative aspirin to achieve the best treatment recovery, then the specific perioperative complications of joint replacement patients in the preoperative aspirin treatment plan will be a feasible method to reduce the potential risks and potential risks in the future.

### Supplementary Information

**Additional file 1.** ICD-9 codes and ICD-10 codes for perioperative complications.

## Data Availability

Data and materials will be available on reasonable request.

## Abbreviations

SA: Shoulder arthroplasty
L-AU: Long-term aspirin use
NIS: National inpatient sample
LOS: Length of stay
AOR: Adjusted odds ratios
UOR: Unadjusted odds ratios
CI: Confidential interval
NIS: Nationwide inpatient sample
CVD: Cardiovascular disease
GI: Gastrointestinal
TXA2: Produces thromboxane A2
PGI2: Prostaglandin I2
