# Supplementary material for: The impact of long-term aspirin use on the patients undergoing shoulder arthroplasty
Source: J Orthop Surg Res. 2023 Nov 23;18:894. doi: 10.1186/s13018-023-04374-4 (PMC10666390; doi:10.1186/s13018-023-04374-4)
Supplement: Supplementary file 1 — Additional file 1. ICD-9 codes and ICD-10 codes for perioperative complications. [file 13018_2023_4374_MOESM1_ESM.docx]

ICD-9^a^ codes and ICD-10^b^ codes for perioperative complications.

| **Category** | **ICD-9 codes** | **ICD-10 codes** |
| --- | --- | --- |
| Blood transfusion | 99.00, 99.02, 99.03, 99.04, 99.05, 99.07, 99.08, V58.2 | 30240N0, 30240N1, 30243H0, 30243H1, 30243N0, 30243N1, 30250H0, 30250H1, 30250N0, 30250N1, 30253H0, 30253H1, 30253N0, 30253N1, 30260H0, 30260H1, 30260N0, 30260N1, 30263H0, 30263H1, 30263N0, 30263N1, 30273H1, 30273N1, 30277H1, 30277N1 |
| Periprosthetic joint infection  (PJI) | 996.66, 996.67, 998.5, 998.51, 998.59 | T81.4, T81.4XXA, T81.4XXD, T81.4XXS, T84.50, T84.50XA, T84.50XD, T84.50XS, T84.59, T84.59XA, T84.59XD, T84.59XS |
| Dislocation of prosthetic joint | 996.42 | T84.028, T84.028A, T84.028D, T84.028S |
| Hemorrhage, seroma, hematoma | 998.1, 998.11, 998.12, 998.13, 729.92, 719.15, 719.16 | I97.42, I97.62, I97.620, I97.621, I97.622, M96.81, M96.810, M96.811, M96.83, M96.830, M96.831, M96.84, M96.84, M96.840, M96.841, M96.842, M96.843, J95.6, J95.61, J95.62, J95.83, J95.830, J95.831, J95.86, J95.860, J95.861, J95.862, J95.863, N99.62, L76.02, K91.841, E89.811, G97.52 |
| Urinary tract infection (UTI) | 590, 590.1, 590.2, 590.3, 590.9, 595, 595.0, 595.3, 595.4, 595.8, 595.9, 597.0, 597.8, 599.0, 997.5 | N39.0, N30.0, N30.00, N30.01, N303, N30.30, N30.31, N308, N30.80, N30.81, N30.9, N30.90, N30.91, N34, N34.0, N34.1, N34.2, N34.3, T83.021, T83.021A, T83.021D, T83.021S |
| Acute renal failure (ARF) | 584, 584.5, 584.6, 584.7, 584.8, 584.9 | N17, N17.0, N17.1, N17.2, N17.8, N17.9 |
| Thrombocytopenia | 287.4, 287.5 | D69.51, D69.59, D69.6 |
| Acute postoperative pain | 338.18 | G89.18 |
| Respiratory disease | 997.3, 997.31, 997.32, 518.51, 518.52, 518.53 | J95.0, J95.00, J95.01, J95.02, J95.03, J95.04, J95.09, J95.2, J95.3, J95.4, J95.61, J95.62, J95.7, J95.71, J95.72, J95.81, J95.811, J95.812, J95.82, J95.821, J95.822, J95.83, J95.830, J95.831, J95.85, J95.850, J95.851, J95.859, J95.86, J95.860, J95.861, J95.862, J95.863, J95.88, J95.89, T81.82, T81.82XA, T81.82XD, T81.82XS |
| Genitourinary disease | 580.0, 580.4, 580.81, 580.89, 580.9, 581.0, 581.1, 581.2, 581.3, 581.81, 581.89, 581.9, 584.5, 584.6, 584.7, 584.8, 584.9, 588.0, 588.1, 588.81, 588.89, 588.9, 590.00, 590.01, 590.10, 590.11, 590.2, 590.3, 590.81, 590.80, 590.9, 595, 595.0, 595.2, 595.3, 595.4, 595.81, 595.82, 5995.89, 595.9, 597.0, 597.8, 597.81, 597.89, 599.0, 997.5, 599.9, 593.89, 593.9, 596.53, 596.6, 596.8, 596.9 | N00, N00.0, N00.1, N00.2, N00.3, N00.4, N00.5, N00.7, N00.8, N00.9, N04.0, N04.1, N04.2, N04.3, N04.4, N04.5, N04.7, N04.8, N04.9, N10, N17, N17.0, N17.1, N17.2, N17.8, N17.9, N25, N25.0, N25.1, N25.8, N25.81, N25.89, N25.9, N30.0, N30.00, N30.01, N30.3, N30.30, N30.31, N30.8, N30.80, N30.81, N30.9, N30.90, N30.91, N34, N34.0, N34.1, N34.2, N34.3, N99.0, N99.1, N998, N99.81, N99.821 |
| Pneumonia | 480, 480.0, 480.1, 480.2, 480.3, 480.8, 480.9, 481, 482, 482.0, 482.1, 482.2, 482.3, 482.30, 482.31, 482.32, 482.39, 482.4, 482.41, 482.42, 482.49, 482.8, 482.81, 482.82, 482.83, 482.84, 482.89, 482.9, 483, 483.0, 483.1, 483.8, 484, 484.1, 484.3, 484.5, 484.6, 484.7, 484.8, 485, 486, 487, 507 | J12.0, J12.1, J12.2, J12.3, J12.81, J12.82, J12.89, J12.9, J13, J14, J15.0, J15.1, J15.20, J15.211, J15.212, J15.29, J15.3, J15.4, J15.5, J15.6, J15.7, J15.8, J15.9, J16.0, J16.8, J17, J18.0, J18.1, J18.2, J18.8, J18.9, J67.8, J67.9, J95.4 |
| Gastrointestinal complication | 997.4 | K91.0, K91.1, K91.2, K91.3, K91.30, K91.31, K91.32, K91.72, K91.81, K91.82, K91.83, K91.841, K91.85, K91.850, K91.858, K91.871, K91.873, K91.89 |
| Convulsion | 781.0, 780.31, 780.32, 780.33, 780.39 | R56, R560, R56.00, R56.01, R56.1, R56.9 |
| Deep venous thrombosis (DVT) | 451, 451.0, 451.1, 451.11, 451.19, 451.2, 451.8, 451.81, 451.82, 451.83, 451.84, 451.89, 451.9, 453.4, 453.40, 453.41, 453.42, 453.8, 453.81, 453.82, 453.83, 453.84, 453.85, 453.86, 453.87, 453.89 | I82.21, I82.210, I82.211, I82.22, I82.220, I82.221, I82.29, I82.290, I82.291, I82.3, I82.40, I82.401, I82.402, I82.403, I82.409, I82.41, I82.411, I82.412, I82.413, I82.419, I82.42, I82.421, I82.422, I82.423, I82.429, I82.43, I82.431, I82.432, I82.433, I82.439, I82.44, I82.441, I82.442, I82.443, I82.449, I82.49, I82.491, I82.492, I82.493, I82.499, I82.4Y, I82.4Y1, I82.4Y2, I82.4Y3, I82.4Y9, I82.4Z, I82.4Z1, I82.4Z2, I82.4Z3, I82.4Z9, I82.62, I82.621, I82.622, I82.623, I82.629 |
| Wound infection | 998.5, 998.51, 998.59, 998.83 | T81.40, T81.4XXA, T81.4XXD, T81.4XXS |
| Pulmonary embolism (PE) | 415.1, 415.11, 415.12, 415.13, 415.19, 415.9 | I260, I26.01, I26.02, I26.09, I26.90, I26.92, I26.93, I26.94, I26.99 |
| Acute myocardial infarction (AMI) | 410.00, 410.01, 410.02, 410.10, 410.11, 410.12, 410.20, 410.21, 410.22, 410.30, 410.31, 410.32, 410.40, 410.41, 410.42, 410.50, 410.51, 410.52, 410.60, 410.61, 410.62, 410.70, 410.71, 410.72, 410.80, 410.81, 410.82, 410.90, 410.91, 410.92, 997.1 | I21, I21.0, I21.01, I21.02, I21.09, I21.1, I21.11, I21.19, I21.2, I21.21, I21.29, I21.3, I21.4, I21.9, I21.A, I21.A1, I21.A9 |
| Peripheral vascular disease | 440.0, 440.1, 440.2, 440.30, 440.31, 440.32, 440.4, 440.8, 440.9, 441.00, 441.01, 441.02, 441.03, 441.1, 441.2, 441.3, 441.4, 441.5, 441.6, 441.7, 441.9, 442.0, 442.1, 442.3, 442.81, 442.82, 442.83, 442.84, 442.89, 442.9, 443.0, 443.1, 443.21, 443.22, 443.23, 443.24, 443.29, 443.81, 443.82, 443.89, 443.9, 444.0, 444.1, 444.21, 444.22, 444.81, 444.89, 444.9, 446.0, 446.1, 446.20, 446.21, 446.29, 446.3, 446.4, 446.5, 446.6, 446.7, 447.0, 447.1, 447.2, 447.3, 447.4, 447.5, 447.6, 447.8, 447.9, 449, 448.0, 448.1, 448.9 | I70.xx-I79.xx |
| Postoperative delirium (POD) | 293, 293.0, 293.1, 293.8, 293.9, 293.81, 293.82, 293.83, 293.84, 293.89, 292.81, 780.97 | F05, F06.0, F06.2, R41.82 |
| Septicemia | 995.91, 995.92 | A02.1, A20.7, A22.7, A40, A40.0, A40.1, A40.3, A40.8, A40.9, A41.01, A41.02, A41.1, A41.2, A41.3, A41.4, A41.50, A41.51, A41.52, A41.53, A41.59, A418, A41.81, A41.89, A41.9 |
| Acute cerebrovascular disease (ACD) | 430.0, 431.0, 432.0, 432.1, 432.9, 433.00, 433.01, 433.10, 433.20, 433.21, 433.30, 433.31, 433.80, 433.81, 433.90, 433.91, 434.00, 434.01, 434.10, 434.11, 434.90, 434.91, 435.0, 435.1, 435.2, 435.3, 435.8, 435.9, 436, 437.2, 437.7 | I60.x, I61.0, I61.1, I61.2, I61.3, I61.4, I61.5, I61.6, I61.8, I61.9, I62.00, I62.01, I62.02, I62.03, I62.1, I62.9, 163.xx, I65.01, I65.02, I65.03, I65.09, I65.1, I65.21, I65.22, I65.23, I65.29, I65.8, I65.9, I66.01, I66.02, I66.03, I66.09, I66.11, I66.12, I66.13, I66.19, I66.21, I66.22, I66.23, I66.29, I66.3, I66.8, I66.9, I67.4, I67.81, I67.82, I67.841, I67.848 |
| Cardiac arrest | 427.5 | I46.2, I46.8, I46.9, I97.71, I97.121, I97.711 |
| Postoperative shock | 998.00, 998.01, 998.02, 998.09 | T81.10, T81.10XA, T81.10XD, T81.10XS, T81.11, T81.11XA, T81.11XD, T81.11XS, T81.12, T81.12XA, T81.12XD, T81.12XS, T81.19, T81.19XA, T81.19XD, T81.19XS |
| Gastrointestinal bleeding  (GI bleeding) | 578.0, 578.1, 578.9 | K91.841, K91.871, K91.873, K25.0, K25.1, K25.2, K25.4, K25.5, K25.6, K26.0, K26.1, K26.2, K26.4, K26.6, K27.0, K27.2, K27.4, K27.6, K28.0, K28.2, K28.4, K28.6, K92.2 |
| stroke | 430, 431, 432, 432.0, 432.1, 432.9, 433, 433.0, 433.00, 433.01, 433.1, 433.10, 433.11, 433.2, 433.20, 433.21, 433.3, 433.30, 433.31, 433.8, 433.80, 433.81, 433.9, 433.90, 433.91, 434, 434.0, 434.00, 434.01, 434.1, 434.10, 434.11, 434.9, 434.90, 434.91, 435, 435.0, 435.1, 435.2, 435.3, 435.8, 435.9, 997.02 | I60.x, I61.0, I61.1, I61.2, I61.3, I61.4, I61.5, I61.6, I61.8, I61.9, I62.00, I62.01, I62.02, I62.03, I62.1, I62.9, 163.X, I65.01, I65.02, I65.03, I65.09, I65.1, I65.21, I65.22, I65.23, I65.29, I65.8, I65.9, I66.01, I66.02, I66.03, I66.09, I66.11, I66.12, I66.13, I66.19, I66.21, I66.22, I66.23, I66.29, I66.3, I66.8, I66.9 |

^a^ ICD-9 refers to the International Classification of Diseases, 9th revision.

^b^ ICD-10 refers to the International Classification of Diseases, 10th revision.
